# Selection and Validation of Stable Reference Genes for RT-qPCR in *Diaphorencyrtus aligarhensis*—A Predominant Parasitoid of *Diaphorina citri*

**DOI:** 10.3390/ijms27114997

**Published:** 2026-05-30

**Authors:** Xiaohang Gu, Bingrui Luo, Siyi Zhang, Jialiang Chen, Peiping Xu, Shuang Li, Baoli Qiu, Lihe Zhang

**Affiliations:** 1 Engineering Research Center of Biotechnology for Active Substances, Ministry of Education, Chongqing Normal University, Chongqing 401331, China; xiaohang2202@outlook.com (X.G.); 2024110513046@stu.cqnu.edu.cn (B.L.); 18996351137@163.com (S.Z.); 13883407923@163.com (J.C.); xupeiping@stu.scau.edu.cn (P.X.); 2 Fruit Research Institute, Chongqing Academy of Agricultural Sciences, Chongqing 401329, China; lishuang@cqaas.cn

**Keywords:** *Diaphorencyrtus aligarhensis*, RT-qPCR analysis, reference genes, *RefFinder*, gene expression

## Abstract

*Diaphorencyrtus aligarhensis* parasitizes the Asian citrus psyllid (ACP), *Diaphorina citri*, the primary insect vector responsible for transmitting Huanglongbing (HLB), a severe citrus disease. Screening of appropriate reference genes is a critical prerequisite for reliable RT-qPCR analysis, which is essential for investigating the functions of target genes in *D. aligarhensis* across diverse experimental conditions. However, to date, no validated reference genes have been reported for this species. This study assessed seven housekeeping genes in *D. aligarhensis* under six conditions (developmental stage, body tissue, population, temperature, diet, and starvation) using five stability algorithms (*geNorm*, *BestKeeper*, *NormFinder*, *RefFinder*, and ∆*Ct*). The results identified the most suitable reference genes for specific experimental conditions: *EIF5A* and *RPL32* for the developmental stage; *RPL13* and *H3* for population comparisons; *RPS6* and *GAPDH* for different feeding diets; *RPL32* and *RPS6* for starvation; *RPL7A* and *RPS6* for different body tissues (head, thorax, abdomen) and temperature gradients (5 °C, 15 °C, 25 °C, 35 °C). Furthermore, the expression profiles of *HSP70* were markedly different when normalized to the most versus the least stable reference genes across body tissues, diets, starvation durations, and temperatures. Our findings establish the first set of RT-qPCR reference genes for *D. aligarhensis*, providing a useful foundation for functional genomics research on this biological control agent.

## 1. Introduction

*Diaphorencyrtus aligarhensis* (Hymenoptera, Encyrtidae) is the dominant endoparasitoid wasp of the Asian citrus psyllid (ACP) *Diaphorina citri*, which is the key insect vector of Huanglongbing (HLB). This parasitoid can reproduce via parthenogenesis or sexual reproduction [1,2,3], and its parasitic behavior shows a significant preference for 2nd to 4th instar nymphs of *D. citri* [4,5,6]. The fecundity of *D. aligarhensis* can reach more than 180 eggs per female, while the developmental cycle of one generation is approximately 15 days, and the average adult longevity is 26 days. There are close physiological and ecological interactions with the host ACP nymphs at each developmental stage [3,4,7,8]. The biological control mechanism of *D. aligarhensis* against ACP is dual: adult females cause immediate ACP mortality by directly feeding on 1st- to 4th-instar nymphs, whereas eggs and larvae cause nutrient depletion and tissue destruction by invading the host hemolymph, eventually leading to the death of the ACP host [9,10]. For decades, it has been used as a biological control agent against *D. citri* worldwide [6,7,8,11].

At present, research on *D. aligarhensis* mainly focuses on its biological characteristics and rarely examines its functional genes and their relationship with *D. citri* [4,5,8]. Gene expression analysis in *D. aligarhensis* plays a vital role in research ranging from functional genomics to host-parasitoid interaction studies. To explore the function of target genes in *D. aligarhensis* and employ this endoparasitoid wasp to control *D. citri* more effectively, it is necessary to establish an appropriate RT-qPCR protocol for subsequent functional genomics studies [12,13,14]. Quantitative real-time polymerase chain reaction (RT-qPCR) is a common method for gene expression analysis [15,16]. Normalization of RT-qPCR data is essential for accurate gene quantification. The most common normalization method involves comparing the mRNA levels of target genes with those of stably expressed reference genes [17]. These reference genes serve to correct and standardize RT-qPCR data, addressing various experimental process-induced variations and, by correcting and standardizing internal experimental differences, ensuring data accuracy and reliability [18].

In a study of *Dialeurodes citri*, *RPS18* and *RPL13* were identified as the most stable reference genes across different temperature conditions. In contrast, across developmental stages, *V-ATP-A* and *RPS18* were identified as the most stable genes [12]. In a study of different developmental stages of the parasitic wasp *Tetrastichus hagenowii*, *α-TUB* showed the best stability and was suitable as an internal reference gene for this stage [14]. Similarly, *α-TUB* also showed strong stability in different developmental stages of *Drosophila suzukii* [19] and *Mythimna separate* [20]. In the parasitoid wasp *Cotesia chilonis*, *RPL17* and *RPL10* were identified as the best reference genes under low temperatures, whereas *18S rRNA*, *histone 3*, and *actin* were more suitable for gene expression analysis under high temperatures [21]. In studies on the reference genes of *Sclerodermus guani*, *SRSF7* and *RPL7A* were found to be the most stable genes across developmental stages, whereas *SRSF7* and *Hsc70* were the most stable reference genes for diet [22]. Therefore, to ensure the accuracy of RT-qPCR, it is crucial to systematically assess reference gene stability across a range of species and conditions to identify the most appropriate reference gene(s).

The lack of identified reference genes in *D. aligarhensis* presents a major constraint, limiting progress in its logical, functional genomics, and proteomics studies. It is crucial to select the appropriate internal reference genes to ensure accurate RT-qPCR results. Therefore, we selected seven generally used reference genes (*RPL7A*, *RPL13*, *RPS6*, *RPL32*, *H3*, *GAPDH*, and *EIF5A*) from the wasp transcriptome database to evaluate their applicability under six conditions (developmental stages, body tissue, population, diet, starvation, and temperature) in *D. aligarhensis*. To determine expression stability, we used five statistical algorithms: ∆*Ct*, *geNorm*, *NormFinder*, *BestKeeper*, and *RefFinder*. Our study provides a set of candidate reference genes for the expression of quantitative functional genes in *D. aligarhensis*, representing the first evaluation of this species.

## 2. Results

### 2.1. PCR Amplification and Performance of Candidate Reference Genes in D. aligarhensis

**Figure 1:**
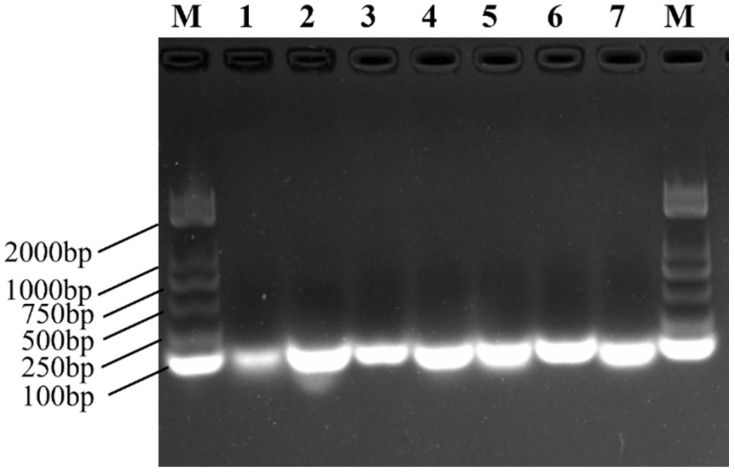
Analysis of PCR products for the seven candidate reference genes by agarose gel electrophoresis. M, DNA marker. The templates in the PCR reactions were as follows: (1) *RPL7A*, (2) *RPL13*, (3) *RPS6*, (4) *RPL32*, (5) *H3*, (6) *GAPDH*, and (7) *EIF5A*.

**Figure 2:**
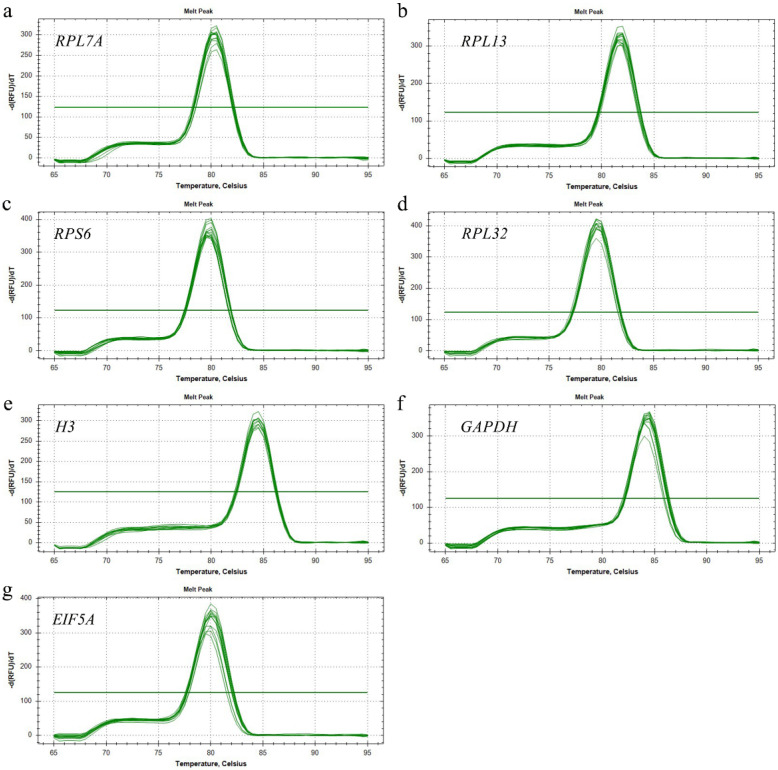
Melting curves of the seven candidate reference genes in *D. aligarhensis*. (**a**) *RPL7A*; (**b**) *RPL13*; (**c**) *RPS6*; (**d**) *RPL32*; (**e**) *H3*; (**f**) *GAPDH*, and (**g**) *EIF5A*.

**Figure 3:**
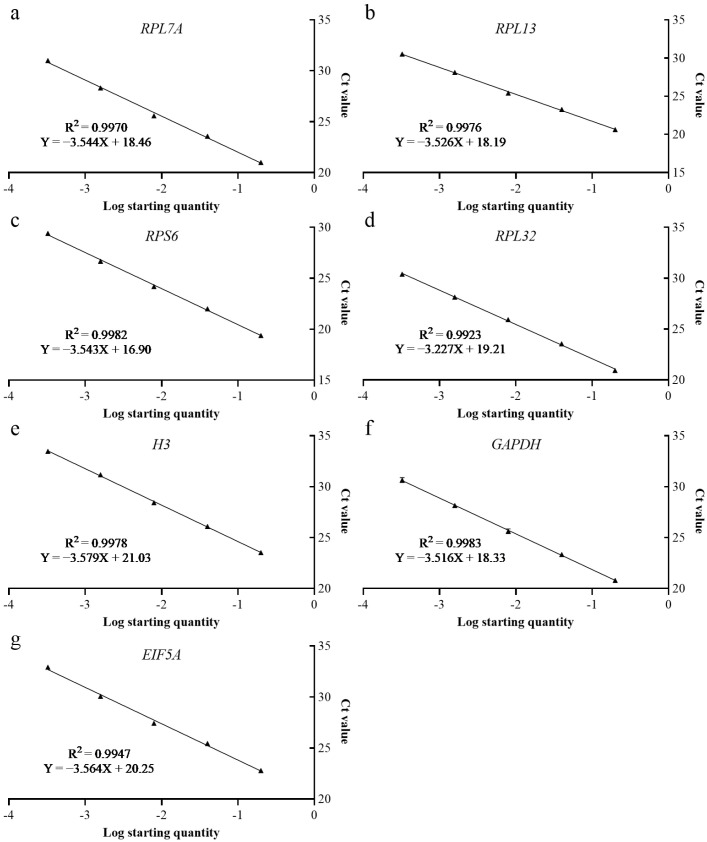
Standard curves of the seven candidate reference genes in *D. aligarhensis*. (**a**) *RPL7A*; (**b**) *RPL13*; (**c**) *RPS6*; (**d**) *RPL32*; (**e**) *H3*; (**f**) *GAPDH*, and (**g**) *EIF5A*.

All seven reference genes were detected in *D. aligarhensis*, each yielding a single band without any non-specific PCR products (Figure 1). The amplified product sizes ranged from 81 bp (*EIF5A*) to 119 bp (*H3*) (Table 1). The RT-qPCR melting curve analysis showed a single peak for each gene, indicating the specificity and stability of the reference genes (Figure 2). Table 1 presents the PCR primer amplification efficiency (E) and correlation coefficient (R^2^) of the seven candidate reference genes, along with the linear regression equations for primer amplification. The standard curves for all seven reference genes are presented in Figure 3. Analysis of the standard curves revealed amplification efficiencies (E) ranging from 90.29% (*H3*) to 104.12% (*RPL32*) (Table 1), and regression coefficients (R^2^) ranging from 0.9326 (*RPL32*) to 0.9983 (*GAPDH*) (Table 1).

### 2.2. Transcript Abundance of the Seven Reference Genes

**Figure 4:**
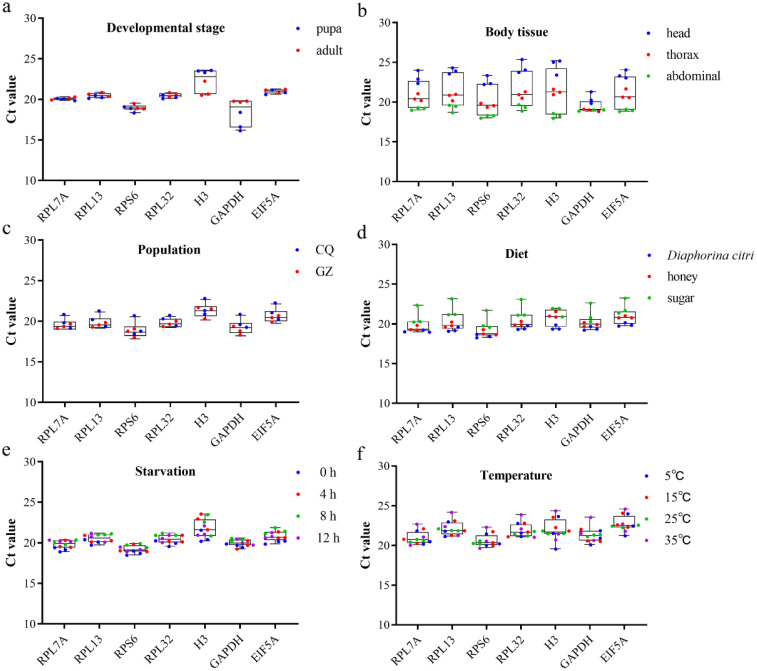
Expression profiles of seven reference genes in six experiments on *D. aligarhensis*. The expression levels of the reference genes are shown as cycling threshold (Ct) values for each experimental condition. (**a**) developmental stage, (**b**) body tissue, (**c**) population, (**d**) diet, (**e**) starvation, and (**f**) temperature.

We quantified the expression of seven candidate reference genes under six experimental conditions using RT-qPCR, based on Ct values. The results indicated Ct values ranging from 16.20 to 25.20 among the candidate reference genes, with *GAPDH* exhibiting the lowest Ct value, suggesting the highest expression level across all experimental conditions. The Ct values for the other candidate housekeeping genes varied between 18.72 and 25.20. As shown in Figure 4, *GAPDH* had the lowest Ct value among the candidate reference genes, indicating a high expression abundance across all experimental conditions. Nonetheless, a comprehensive analysis is necessary to ascertain its suitability as an internal reference gene under different conditions.

### 2.3. Stability of the Reference Genes Under Specific Experimental Conditions

We determined the most reliable reference gene across all experimental conditions in *D. aligarhensis* using five algorithms: *geNorm*, *NormFinder*, *BestKeeper*, ∆*Ct*, and *RefFinder*.

It should be noted that different stability algorithms may yield slightly different rankings for the same dataset, as each method applies a different mathematical definition of expression stability (pairwise variation, variance modeling, standard deviation of Ct values, etc.) [23,24,25,26,27]. Such discrepancies are common in reference gene screening studies and highlight the importance of using a consensus approach, such as *RefFinder*, in combination with experimental validation of target genes.

#### 2.3.1. *geNorm* Method

**Figure 5:**
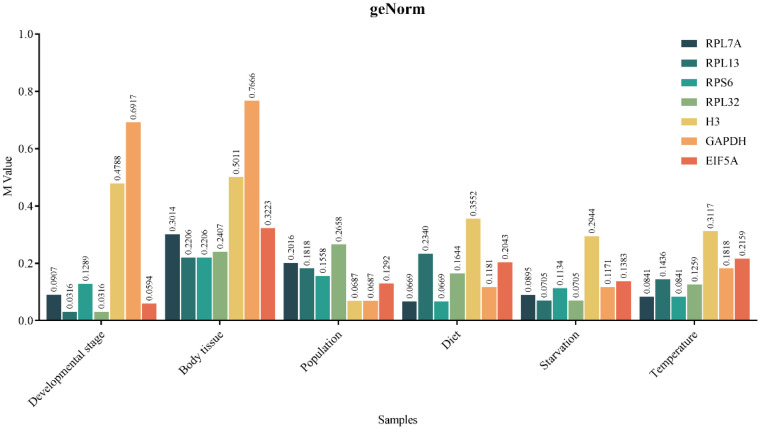
Expression stability of candidate reference genes was analyzed using *geNorm*.

The stability of candidate reference genes across the various treatment groups was assessed using *geNorm*, which calculates the stability measure value (*M*) for each gene. A lower *M* value indicates a higher stability of the reference gene [23,24]. Analysis revealed that *RPL13* and *RPL32* were the most stable candidate reference genes across developmental stages and starvation treatment groups. Similarly, *RPL13* and *RPS6* were the most stable genes in different body tissues, whereas *H3* and *GAPDH* were the most stable genes among various geographical populations. In contrast, *RPL7A* and *RPS6* were the most stable genes across the different dietary and temperature groups (Figure 5).

**Figure 6:**
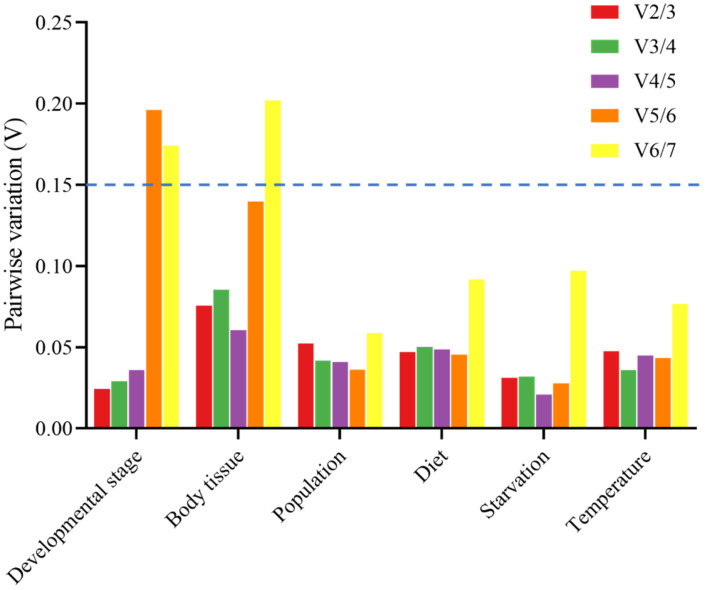
Pairwise variation (V) values were calculated across six experimental conditions: developmental stage, body tissue, population, diet, starvation, and temperature. A Vn/Vn + 1 value ≤ 0.15 indicates that n reference genes are sufficient for reliable normalization.

The *geNorm* algorithm was used to determine the optimal number of reference genes by calculating the pairwise variation (V). As all V-values (V2/3) were below the 0.15 threshold, two reference genes were sufficient for reliable normalization across all experimental conditions in this study. (Figure 6).

#### 2.3.2. *NormFinder* Method

**Figure 7:**
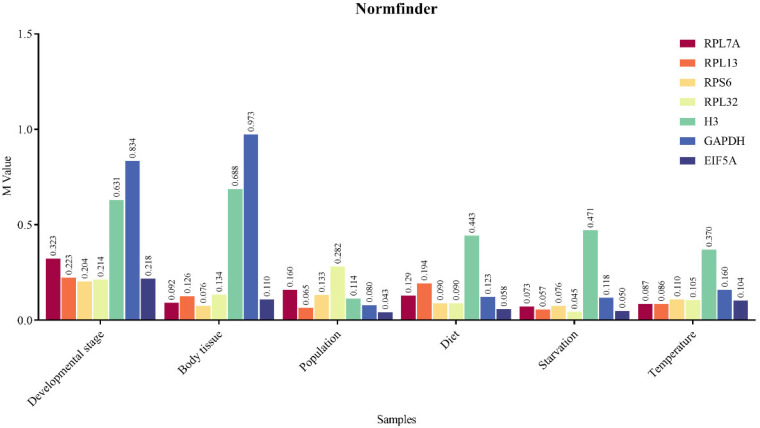
Expression stability of candidate reference genes was analyzed using *NormFinder*.

*NormFinder* software measures gene expression stability by evaluating the stability value (*M* value) of each candidate reference gene. A lower M value indicates a higher stability of the reference gene [24,25]. Based on *NormFinder* analysis, *RPS6* was identified as the most stable gene across all developmental stages and body tissues. *EIF5A* showed the most stable expression across population and diet comparisons. Furthermore, during starvation stress, *RPL32* was the most stable reference gene, whereas *RPL13* showed the most consistent expression in samples exposed to different temperatures (Figure 7).

#### 2.3.3. *BestKeeper* Method

*BestKeeper* software performed a pairwise correlation analysis of Ct values for candidate reference genes within the sample groups. A set of descriptive statistics (including the standard deviation (SD), coefficient of variation (CV), and pairwise correlation coefficient (Pearson’s r)) was derived for the gene set. The stability of the reference gene was evaluated according to the SD. The gene with the lowest SD was the most stable reference gene, and genes with SD < 1 were considered acceptable [24,26].

The dataset analysis revealed that *H3* and *GAPDH* had SD values exceeding 1.0 across developmental stages, excluding them as reference genes. Similarly, *RPL13* was unsuitable as a reference gene for different dietary conditions. In contrast, the seven candidate reference genes were appropriate for different geographical populations, starvation durations, and temperature conditions. Notably, *GAPDH* was the only suitable reference gene for different body tissues (Table 2).

#### 2.3.4. ∆*Ct* Method

∆*Ct* identifies stable reference genes by assessing the relative expression of “gene pairs” formed from candidate reference genes within a sample. If the ∆*Ct* value between the two genes remains constant in the same sample, their expression is deemed stable. Thus, the ∆*Ct* method selects the reference gene with the lowest average standard deviation of ∆*Ct* among all gene pairs as the most stable reference gene [27,28].

**Figure 8:**
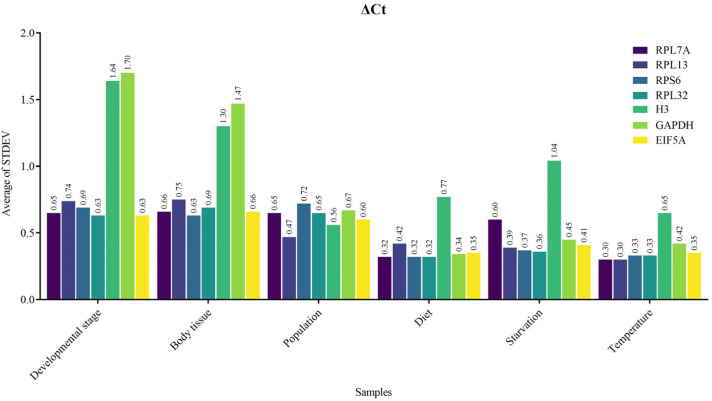
Expression stability of candidate reference genes analyzed using ∆*Ct*.

As shown in Figure 8, *RPL32* and *EIF5A* had the smallest SD values across developmental stages, indicating greater stability in comparisons between developmental stages and making them the most stable reference genes. Similarly, *RPL7A* and *RPL13* were the most stable genes under varying temperature conditions. *RPS6*, *RPL7A*, and *RPL32* were the optimal reference genes for the different dietary conditions. However, only one stable reference gene was identified across different populations, body tissues, and starvation conditions: *RPL13*, *RPS6*, and *RPL32*, respectively.

### 2.4. Stability Ranking of the Seven Reference Genes

To avoid single-software analysis errors, *RefFinder* was used to comprehensively assess the stability of each candidate reference gene. This tool can calculate the geometric mean of the ranking values for each candidate reference gene, obtained with *geNorm, NormFinder*, *BestKeeper*, and ∆*Ct* software, to produce a comprehensive ranking by directly entering Ct values online [29].

**Figure 9:**
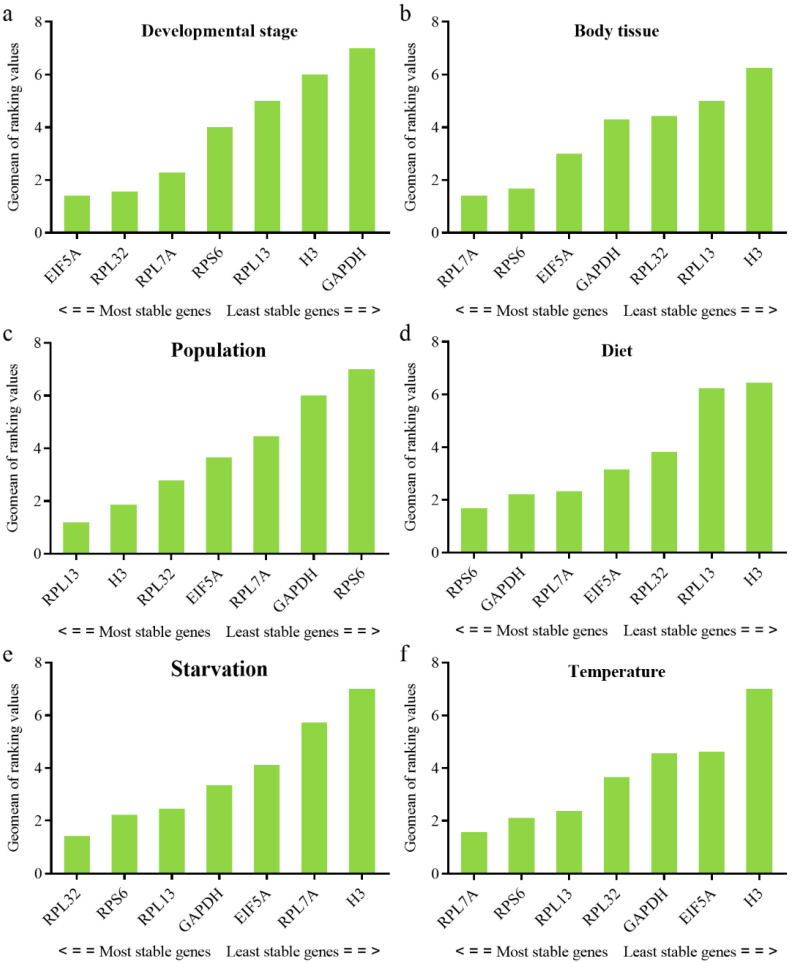
Expression stability was ranked using *RefFinder*, with lower geometric mean values indicating greater stability. (**a**) Developmental stage, (**b**) body tissue, (**c**) population, (**d**) diet, (**e**) starvation, and (**f**) temperature.

Because these four algorithms employ different mathematical definitions of expression stability (pairwise variation, variance modeling, standard deviation of Ct values, etc.), minor discrepancies among their individual rankings (as seen in Figure 5, Figure 7 and Figure 8 and Table 2) are expected and do not invalidate the consensus results. The final recommendations for each experimental condition were therefore based on the *RefFinder* rankings, and their reliability was further supported by experimental validation using the target gene *HSP70* (Section 2.5). The comprehensive stability rankings for each condition are presented in Figure 9.

Based on the *RefFinder* analysis, the comprehensive ranking of reference gene stability across developmental stages was as follows: *EIF5A* > *RPL32* > *RPL7A* > *RPS6* > *RPL13* > *H3* > *GAPDH* (Figure 9a). The comprehensive stability ranking of different body tissues, from high to low, was as follows: *RPL7A* > *RPS6* > *EIF5A* > *GAPDH* > *RPL32* > *RPL13* > *H3* (Figure 9b). Similarly, the stability comprehensive ranking of different geographical populations was *RPL13* > *H3* > *RPL32* > *EIF5A* > *RPL7A* > *GAPDH* > *RPS6* (Figure 9c). For the different dietary groups, the ranking was as follows: *RPS6* > *GAPDH* > *RPL7A* > *EIF5A* > *RPL32* > *RPL13* > *H3* (Figure 9d). Comparing different starvation durations, we observed that the comprehensive stability ranking was *RPL32* > *RPS6* > *RPL13* > *GAPDH* > *EIF5A* > *RPL7A* > *H3* (Figure 9e); however, the recommended comprehensive ranking of candidate reference genes across different temperature treatment groups was as follows: *RPL7A* > *RPS6* > *RPL13* > *RPL32* > *GAPDH* > *EIF5A* > *H3* (Figure 9f).

### 2.5. Validation of Reference Gene Stability Using HSP70

**Figure 10:**
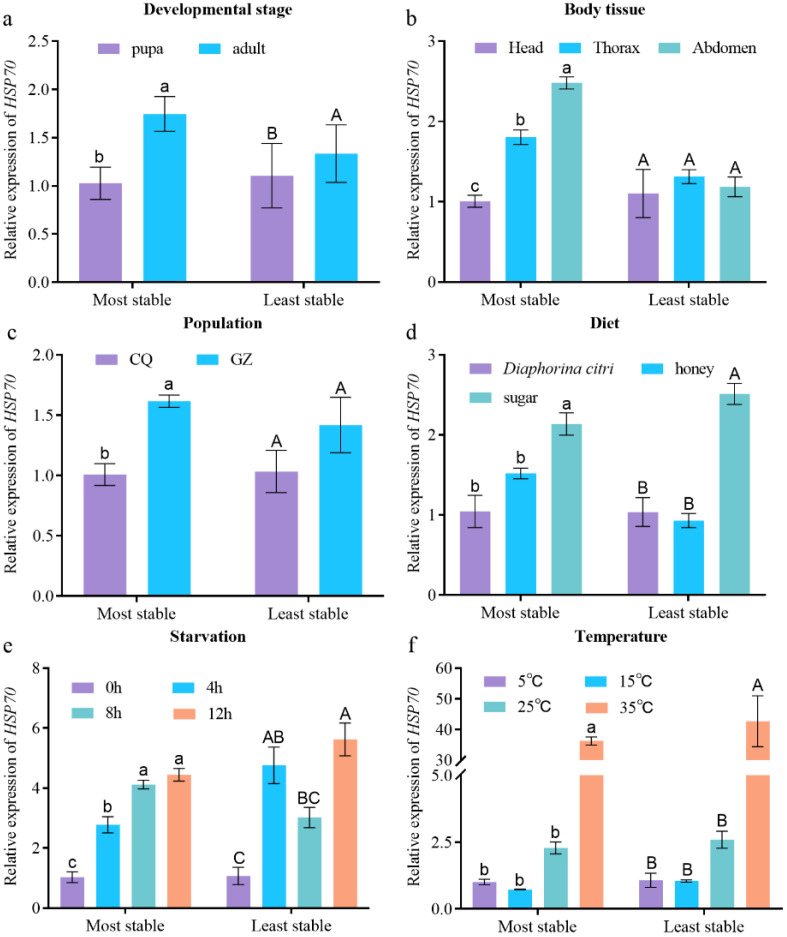
Relative expression of *HSP70* was normalized to optimal and least-stable reference genes under different experimental conditions. (**a**) Developmental stages using *EIF5A* and *RPL32* (most stable) and *H3* and *GAPDH* (least stable); (**b**) body tissues using *RPL7A* and *RPS6* (most stable) and *RPL13* and *H3* (least stable); (**c**) populations using *RPL13* and *H3* (most stable) and *GAPDH* and *RPS6* (least stable); (**d**) diets using *RPS6* and *GAPDH* (most stable) and *RPL13* and *H3* (least stable); (**e**) starvation duration using *RPL32* and *RPS6* (most stable) and *RPL7A* and *H3* (least stable); (**f**) temperature using *RPL7A* and *RPS6* (most stable) and *EIF5A* and *H3* (least stable). Values are expressed as the mean ± standard error. Different lowercase letters indicate significant differences from the most stable gene (*p* < 0.05). Different uppercase letters indicate significant differences when normalized to the least stable (*p* < 0.05).

To accurately evaluate and compare the stability of the seven candidate reference genes, we used the relative expression levels of *HSP70* (a target gene) to validate the stability of the selected reference genes under the six experimental conditions. Based on the analysis of the results, we found that the expression patterns and levels of *HSP70* were inconsistent in different body tissues (Figure 10b), diets (Figure 10d), durations of starvation (Figure 10e), and temperatures (Figure 10f), as revealed by comparing the most stable candidate reference genes with the least stable one (except for different developmental stages (Figure 10a) and populations (Figure 10c)).

## 3. Discussion

In this study, we systematically evaluated the stability of seven candidate reference genes under various experimental conditions. Our results provide a basis for selecting appropriate reference genes for RT-qPCR normalization in *D. aligarhensis*, thereby facilitating subsequent functional gene research in this species.

RT-qPCR analysis of target gene expression under different conditions is a commonly used method in molecular biology studies [30,31,32,33]. Reference genes are generally considered to be the most stable genes expressed in all cell types [15,27]; however, there is no “universal” reference gene for stable expression under all possible conditions [19,34,35]. Moreover, over-reliance on a single reference gene in many expression studies risks compromising data accuracy and leading to misleading conclusions [15,36,37]. Therefore, it is important to verify the stability of the selected reference genes under different experimental conditions [12,13]. In recent years, a growing number of studies have screened for reference genes in insects from the orders Hemiptera, Lepidoptera, Coleoptera, and Diptera [12,37,38,39]. However, there are few reports on the evaluation of reference genes in parasitic wasps.

It is important to note that many of the cited studies involve insects that are distantly related to Hymenoptera. While direct cross-species comparisons of absolute stability rankings are of limited value, these examples collectively reinforce a key principle: reference gene suitability cannot be generalized across species or conditions without empirical validation. Therefore, the following comparisons are presented to highlight the diversity of outcomes in reference gene screening, rather than to suggest evolutionary conservation.

Our study found that none of the selected reference genes could serve as a “universal” reference across all six experimental conditions. These results are similar to previous findings in other insects [13,34,39,40,41] and highlight an important principle that reference gene stability must be validated for each species and experimental condition, rather than assumed. Previous studies have emphasized that standardized RT-qPCR requires multiple reference genes, with the optimal number being context dependent. Previous studies emphasize that standardized RT-qPCR requires multiple reference genes, with the optimal number being context-dependent. For instance, normalization in the developmental stages of *Coleomegilla maculata* necessitated at least five genes, whereas sex-specific analysis required only three [37]. For gene expression studies in *Dialeurodes citri*, only two reference genes are needed for developmental stages, populations, and temperature variations; however, three are necessary for reliable normalization across genders [12]. Our *geNorm* analysis of pairwise variation confirmed that the two reference genes were adequate across all experimental conditions, which is consistent with previous studies [13,42].

In our study, ribosomal protein (RP) genes, especially *RPL7A*, *RPS6*, and *RPL32*, were consistently stable across multiple conditions (e.g., body tissue, temperature, starvation, and diet). This is likely because RP genes play essential roles in protein synthesis and require stable expression, regardless of environmental or physiological changes. Their high evolutionary conservation also makes them good reference gene candidates, as reported for many other insects [18,43,44]. In contrast, *GAPDH*, a commonly used housekeeping gene, was stable only under dietary conditions in our experiments and showed high variability under temperature- and developmental-stage treatments. One possible reason is that *GAPDH* is involved in stress-responsive metabolic pathways, which may change its expression under certain conditions [45].

Studies on other hymenopteran parasitoids have shown different results. For example, in *Tetrastichus hagenowii*, α-tubulin is the most stable gene across developmental stages [14]. In *Cotesia chilonis*, *RPL17* and *RPL10* were most stable at low temperatures, whereas *18SrRNA* performed better at high temperatures [21]. In *Sclerodermus guani*, *SRSF7* and *RPL7A* were the most stable for developmental stages, and *SRSF7* and *Hsc70* for diet [22]. In our study, *RPL7A* was among the top candidates for body tissue and temperature, whereas *RPL13* was stable only for population comparison. These differences suggest that even among closely related parasitoid wasps, no single reference gene works well under all conditions. Therefore, reference gene selection should be based on a specific experimental design.

Our comprehensive analysis using *RefFinder* (geometric mean of four algorithms) and validation with *HSP70* supports the following recommendations: for the developmental stage, *EIF5A* and *RPL32* were used; for body tissue and temperature, *RPL7A* and *RPS6* were used; for population comparisons, *RPL13* and *H3* were used; for diet, *RPS6* and *GAPDH* were used; and for starvation, *RPL32* and *RPS6* were used. Two reference genes are sufficient for each condition, as indicated by *geNorm* pairwise variation (V < 0.15). Using unstable reference genes (e.g., *H3* and *GAPDH*) across developmental stages can lead to incorrect expression patterns, as demonstrated by *HSP70* (Figure 10). Thus, for future functional studies in *D. aligarhensis*, we strongly recommend using validated reference gene pairs appropriate for each experimental condition.

We acknowledge that only two geographically distinct subpopulations (Guangzhou and Chongqing) were compared in this study. While our results suggest that *RPL13* and *H3* are stably expressed in these two populations, additional field populations are needed to generalize these findings across the entire distribution of the species. Future research should validate the stability of these reference genes across additional populations from diverse ecological regions.

This study comprehensively assessed seven reference genes under six conditions (developmental stage, body tissue, population, diet, starvation, and temperature) using five algorithms (*geNorm*, ∆*Ct*, *BestKeeper*, *NormFinder*, and *RefFinder*). According to the *RefFinder* comprehensive evaluation results, a set of stable reference genes was recommended for each experimental condition in this study. Our results further showed that no universal reference gene was suitable across all conditions. Our work provides a useful reference for designing RT-qPCR experiments in *D. aligarhensis* and will support future functional genomics studies in this species.

The validation of reference gene stability was performed using only one target gene (*HSP70*). Although *HSP70* is sensitive to the tested conditions (temperature, starvation, diet, and tissue) and is therefore suitable for demonstrating the effect of reference gene selection, the inclusion of additional target genes (e.g., a constitutively expressed or developmentally regulated gene) would provide stronger support for the generalizability of our conclusions. Future studies should address this limitation.

## 4. Materials and Methods

### 4.1. Insect Rearing

Two subpopulations of *D. aligarhensis* were used in this study: The Guangzhou subpopulation (GZ) was collected from *D. citri* nymphs on *Murraya exotica* at South China Agricultural University (Guangzhou, Guangdong Province, China) in June 2022, and the Chongqing subpopulation (CQ) was collected from the same host plant species in Chongqing Municipality (Southwest China) in May 2024. Both subpopulations were reared under standard laboratory conditions (26 ± 1 °C, 70 ± 10% R.H., 14L/10D photoperiod) using *D. citri* nymphs as hosts, with the GZ subpopulation maintained at South China Agricultural University and the CQ subpopulation at Chongqing Normal University (Chongqing, China) for multiple generations.

### 4.2. Sample Collection of D. aligarhensis

We evaluated the stability of *D. aligarhensis* reference genes across a range of conditions, including developmental stages, adult female body tissues, different subpopulations, temperature stress, starvation, and varying adult diets. Except for the population comparison experiment, which included both the Guangzhou (GZ) and Chongqing (CQ) subpopulations, all other samples were collected exclusively from the CQ subpopulation. We collected both pupal and adult stages of *D. aligarhensis* in this study The larval stage was excluded because (i) larvae are extremely small (<0.5 mm in length), making it difficult to obtain sufficient RNA yield for reliable RT-qPCR analysis, and (ii) the primary focus of this study and the intended application of these reference genes are on pupal and adult stages, which are the optimal stages for field release in biological control programs. Each biological replicate comprised 30 pupae and 20 adult mosquitoes. To isolate different body tissues, we dissected approximately 40 adult females to collect their heads, thoraxes, and abdomens; a total of 120 individuals were used across three replicates (*n* = 40). We collected 60 females (three biological replicates, *n* = 20) from each of the two subpopulations (South China and Southwest China) for population-level assessment. For the temperature treatments, 240 *D. aligarhensis* female adults were divided into four groups (*n* = 60) and exposed to 5, 15, 25, or 35 °C for 3 hours. The 3-hour exposure time was chosen based on preliminary experiments that showed that this duration induced detectable stress responses without causing acute mortality. For starvation treatment, the adults were starved for 0 h, 4 h, 8 h, and 12 h, respectively, and 20 individuals constituted one replicate. For dietary treatment, adults were reared on a diet of citrus psyllid nymphs, sucrose, and honey. After 10 days, samples were collected, with 20 individuals constituting each replicate. The 10-day feeding period was selected based on preliminary observations indicating that adult wasps reach a physiological steady state after 10 days on a given diet. Because an individual adult *D. aligarhensis* is approximately 1 mm in length and yields very low total RNA (typically <50 ng), a single insect does not provide sufficient RNA for RT-qPCR. Therefore, individuals were pooled into a single biological replicate, and the number of individuals per pool was specified above for each condition. Regarding the exclusive use of females, *D. aligarhensis* exhibits thelytokous parthenogenesis (virgin females produce female offspring), and males are extremely rare in the laboratory colonies. Moreover, in biological control applications, female wasps actively parasitize *D. citri* nymphs, thereby contributing to pest suppression. Therefore, only female rats were used in this study. All experiments were performed in triplicate, and upon collection, samples were immediately snap-frozen in liquid nitrogen and stored at −80 °C until analysis.

Three independent biological replicates were performed for each experimental condition. Each biological replicate consisted of a pool of insects collected from separate rearing cages on different dates to ensure their biological independence. The number of individuals per pool was specified above for each condition.

### 4.3. Candidate Reference Genes and Primer Design

Seven reference genes (*RPL7A*, *RPL13*, *RPS6*, *RPL32*, *H3*, *GAPDH*, and *EIF5A*) that are commonly used for RT-qPCR in other insect species were selected and evaluated. Common housekeeping genes, such as 18SrRNA and alpha-tubulin, were not included. 18SrRNA was depleted during RNA-seq library preparation and was absent from the transcriptome assembly. Alpha-tubulin could not be identified by targeted BLAST (available at https://blast.ncbi.nlm.nih.gov/Blast.cgi/, accessed on 12 February 2025) searches using conserved insect sequences against our de novo transcriptome, likely because of the lack of a reference genome for *D. aligarhensis*. Therefore, only the seven genes mentioned above were selected as the candidates. Primer pairs were designed using Primer Premier 5, based on recently sequenced *D. aligarhensis* transcriptomes (unpublished data; available upon request). To evaluate gene stability, we selected primers that produced a single, specific band of 80–120 bp. The PCR reaction system contained a 25 μL reaction mixture with the 2× SanTaq^TM^ PCR Master Mix (with Blue Dye) (Sangon Biotech, Shanghai, China). PCR was performed according to an established method [12,13,46]. To validate the PCR products, 1% agarose gel electrophoresis was performed at 120 V for 25 min [14].

### 4.4. RNA Isolation and cDNA Synthesis

Total RNA was extracted from each sample using the standard Trizol reagent protocol (Takara, Tokyo, Japan). The total RNA concentration was measured using a NanoDrop One spectrophotometer (Thermo Fisher Scientific, Waltham, MA, USA). The extracted total RNA was resuspended in 20 μL ddH_2_O. For first-strand cDNA synthesis, we used 1 μg of total RNA per sample with the PrimeScript^TM^ RT Kit (Takara, Kyoto, Japan), following the manufacturer’s protocol. The synthesized cDNAs were diluted 10-fold and used for reverse transcription-quantitative PCR (RT-qPCR).

RNA extraction and cDNA synthesis were independently performed for each biological replicate. Each sample pool (as described in Section 4.2) was processed separately, and no pooling of RNA or cDNA across biological replicates was performed.

### 4.5. RT-qPCR Analysis

A total of 50 μL of reaction mixture was prepared, consisting of the following components: 25 μL of TB Green^®^ Premix Ex Taq™ II (Takara, Kyoto, Japan), 2.5 μL of diluted cDNA template, 17.5 μL of RNase-free water, 2.5 μL of forward primer, and 2.5 μL of reverse primer. The mixture was then divided into three technical replicates of 15 μL each for analysis. The Ct values from the three technical replicates were averaged for each biological replicate before further analysis. The standard deviation among technical replicates was consistently <0.2 cycles, indicating high repeatability. The qPCR protocol included an initial denaturation phase at 95 °C for 3 min, followed by 40 amplification cycles alternating between 95 °C for 10 s (denaturation) and 55 °C for 30 s (annealing/extension). Subsequently, melting curve analysis was performed by increasing the temperature from 55 to 95 °C, with a 10-s hold and a 5 °C increment every 10 s [13]. A CFX96 real-time PCR system (Bio-Rad, Hercules, CA, USA) was used for thermal cycling. RT-qPCR quantification of individual genetic targets was performed using slope-based analytical methods and linear regression. Calibration curves for each oligonucleotide primer set were generated by fivefold serial dilutions of cDNA concentrations covering a dynamic range from 5^−1^ to 5^−5^. We determined the RT-qPCR amplification efficiency (*E*) using the standard formula: *E* = [10^(−1/slope) − 1] × 100 [47].

### 4.6. Stability Evaluation of Candidate Reference Genes

We separately analyzed the data from the six experimental conditions. The stability of each candidate reference gene was assessed using various software tools, including *geNorm* (version 3.5) [23], *NormFinder* (version 20) [25], *BestKeeper* (version 1) [26], the ∆*Ct* method [27], and *RefFinder* [29]. To analyze using *geNorm* and *NormFinder*, Ct values were converted as follows: the lowest Ct value of a candidate gene among all samples was identified, and the Ct value of the reference gene in other samples was subtracted to obtain Δ*Ct*, and then the relative expression level of the reference gene in other samples was determined using the formula 2^−Δ*Ct*^ [24]. *RefFinder* (available at http://blooge.cn/RefFinder/, accessed on 22 May 2025) amalgamates the stability rankings of candidate reference genes by calculating the geometric mean of ranking values derived from four distinct analytical methods: *geNorm*, *NormFinder*, *BestKeeper*, and the Δ*Ct* method. This resulted in the generation of a comprehensive stability index. The composite stability ranking index adheres to an inverse-proportionality principle, where lower index values indicate higher transcriptional stability, facilitating the establishment of a hierarchical reliability classification among candidate reference genes.

### 4.7. Validation of Reference Gene Stability Using a Target Gene (HSP70)

To assess the stability of candidate reference genes, we analyzed the expression of the heat shock protein 70 gene (*HSP70*) as a target gene (not a reference gene) in the parasitoid wasp *D. aligarhensis*. The target gene primers were designed as follows: forward, 5′-TCGCTGGCTTGATAATAATACCTTGG-3′ and reverse, 5′-TTGGTCATAATCGGTGAACACTGTC-3′. The relative transcript levels of the *HSP70* gene across experimental conditions were quantified using the 2^−ΔΔ*Ct*^ method with three biological replicates [48].

### 4.8. Statistical Analysis

Three biological replicates were included in each experimental group. The Ct values were imported into Microsoft Excel 2021 to calculate the mean and standard deviation for each gene. To validate reference gene stability, we employed one-way ANOVA followed by Tukey’s test in SPSS 27.0 (SPSS Inc., Chicago, IL, USA) to analyze the significant differences in *HSP70* expression across treatments, and charts were generated using GraphPad Prism 9.5.0 (GraphPad Software, San Diego, CA, USA).

## Figures and Tables

**Table 1:** Candidate reference genes evaluated in this study.

Gene	Primer Sequences (5′–3′)	Length (bp)	Efficiency (%)	R^2^	Linear Regression
*RPL7A*-F	TGTTGCTCTGACACAGGTTGATTC	93	91.50	0.9970	Y = −3.544X + 18.46
*RPL7A*-R	TCATCGTGACGGTCATTGAAGTTG	93			
*RPL13*-F	AACCGTATAAAGAAGCCCGTTCC	114	92.14	0.9976	Y = −3.526X + 18.19
*RPL13*-R	GAATCCTCTGCCTAAGCGTGTC	114			
*RPS6*-F	AGCAAGCCGATGAATACCACAAG	91	91.53	0.9982	Y = −3.543X + 16.90
*RPS6*-R	GCACTACGCTTTCTCTTCAGTTCC	91			
*RPL32*-F	ACGCCATCAAAGTGATCGCTATG	89	104.12	0.9923	Y = −3.227X + 19.21
*RPL32*-R	CCTTGAACCTCCTACGGACTCTG	89			
*H3*-F	TCAAGACCGATCTCCGCTTCC	119	90.29	0.9978	Y = −3.579X + 21.03
*H3*-R	GACACGCTTGGCATGGATGG	119			
*GAPDH*-F	CGCAACTTACGACCAGATCAAGG	116	92.49	0.9983	Y = −3.516X + 18.33
*GAPDH*-R	CACCAACGAAATCCGACGAGAC	116			
*EIF5A*-F	AATGGCTGACAATGGGACTTACG	81	90.80	0.9947	Y = −3.564X + 20.25
*EIF5A*-R	TTGTAGTCAAGACGAAGCTGTACG	81			

**Table 2:** Expression stability of candidate reference genes analyzed using *BestKeeper*.

Genes	Standard Deviation					
	Developmental Stage	Body Tissue	Population	Diet	Starvation	Temperature
*RPL7A*	0.12	1.46	0.59	0.75	0.42	0.65
*RPL13*	0.21	1.74	0.55	1.00	0.44	0.67
*RPS6*	0.24	1.67	0.62	0.75	0.38	0.61
*RPL32*	0.22	1.79	0.48	0.89	0.44	0.67
*H3*	1.16	2.13	0.59	0.81	0.96	0.98
*GAPDH*	1.34	0.63	0.58	0.70	0.33	0.64
*EIF5A*	0.22	1.66	0.60	0.77	0.48	0.73

## Data Availability

The original contributions presented in this study are included in the article. Further inquiries can be directed to the corresponding authors.

## Abbreviations

The following abbreviations are used in this manuscript.

h	Hour
d	Day
L	Light
D	Dark
R^2^	Correlation Coefficient
*RPL7A*	Ribosomal Protein L7a
*RPL13*	Ribosomal Protein L13
*RPS6*	Ribosomal Protein S6
*RPL32*	Ribosomal Protein L32
*H3*	Histone H3
*GAPDH*	Glyceraldehyde-3-phosphate Dehydrogenase
*EIF5A*	Eukaryotic Translation Initiation Factor 5A
*HSP70*	Heat Shock Protein 70
